# Supplementary material for: A UMLS-based spell checker for natural language processing in vaccine safety
Source: BMC Med Inform Decis Mak. 2007 Feb 12;7:3. doi: 10.1186/1472-6947-7-3 (PMC1805499; doi:10.1186/1472-6947-7-3)
Supplement: Additional file 13 — RAP application directory. Sets up directory for RAP (RDF application for PHP) source code [file 1472-6947-7-3-S13.gz › rap/doc/tutorial/usingtheResModelAPI.htm]

Using RAP's Resource-Centric ResModel API


### RDF API for PHP V0.9.1

# Using RAP's Resource-Centric ResModel API

This turorial is part of the RAP - Rdf API for PHP documentation.

Daniel Westphal, Chris Bizer  
October 2004

This tutorial is based on the Jena
tutorial by *Brian McBride*, *Daniel Boothby and Chris Dollin.*

## Table of Contents

1. Introduction
2. Navigating a Model
3. Querying a Model
4. Containers

## Introduction

RAP offers two different programming interfaces for manipulating RDF graphs:
The statement-centric **Model API** which allows you to manipulate
an RDF graph as a set of statements; and the resource-centric **ResModel
API** for manipulating an RDF graph as a set of resources having properties.
The ResModel API is very similar to the Jena
Model API allowing programmers, which are used to Jena, to easily write
RAP code.

The ResModel API is implemented on top of the Model API. ResModels are always
having a underlying MemModel, DbModel, InfModelF or InfModelB and are only providing
a resource-centric view on this model. Each method call is translated into a
find()-, add()-, or remove()-call to the underlying model. Thus using ResModel
is slightly slower than using the underlying model directly.

RDF is a W3C standard for describing resources. What is a resource? That is
rather a deep question and the precise definition is still the subject of debate.
For our purposes we can think of it as anything we can identify. You are a resource,
as is your home page, this tutorial, the number one and the great white whale
in Moby Dick.

Our examples in this tutorial will be about people. They use an RDF representation of VCARDS . RDF is best thought of in the form of node and arc diagrams. A simple vcard might look like this in RDF:

The *resource* , John Smith, is shown as
an ellipse and is identified by a Uniform Resource Identifier (URI), in this
case "http://.../JohnSmith".  
Resources have *properties*. In these examples
we are interested in the sort of properties that would appear on John Smith's
business card. The figure above shows only one property, John Smith's full name.
A property is represented by an arc, labeled with the name of a property. The
name of a property is also a URI, but as URI's are rather long and cumbersome,
the diagram shows it in XML qname form. Each property has a value. In this case
the value is a *literal*, which for now we
can think of as a strings of characters. Literals are shown in rectangles.

In RAP, the classes representing resources, properties and literals are called
ResResource, ResProperty and ResLiteral. The code below creates John's VCard
using the ResModel API::

//change the RDFAPI\_INCLUDE\_DIR to your local settings  
define("RDFAPI\_INCLUDE\_DIR", "C:/!htdocs/rdfapi-php/api/");   
include(RDFAPI\_INCLUDE\_DIR . "RdfAPI.php");

// Some definitions  
define('VCARD\_NS', 'http://www.w3.org/2001/vcard-rdf/3.0#');  
$personURI = "http://somewhere/JohnSmith";  
$fullName = "John Smith";

// Create an empty Model   
$model = ModelFactory::getResModel(MEMMODEL);

// Create the resources  
$fullNameLiteral = $model->createLiteral($fullName);  
$johnSmith = $model->createResource($personURI);  
$vcard\_FN= $model->createProperty(VCARD\_NS.'FN');  
$vcard\_NICKNAME= $model->createProperty(VCARD\_NS.'NICKNAME');

// Add the property   
$johnSmith->addProperty($vcard\_FN, $fullNameLiteral);

The code begins with some constant definitions and then creates an empty model,
using the ModelFactory method getResModel(MEMMODEL) to create a ResModel
using a underlying MemModel. RAP also contains other implementations of models,
e.g. one which uses a relational database, or models that support RDFS reasoning
mechanisms; these types of Model are also available from the ModelFactory.

## Navigating a Model

Given the URI of a resource, the resource object can be retrieved from a model
using the $model->createResource($uri) method. This method is defined to
return a ResResource object if one exists in the model, or otherwise to create
a new one. Example: Retrieve the John Smith resource from the model:

// Retrieve the John Smith vcard resource from the model   
$vCard = $model->createResource($personURI);

The ResResource interface defines a number of methods for accessing the properties
of a resource. The $resource->getProperty($property) method accesses a property
of the resource. The type of the object returned is Statement, not the Property
as you might expect. Returning the whole statement allows the application to
access the value of the property using one of its accessor methods which return
the object of the statement. Example: Retrieve the value of the FN property:

// Retrieve the value of the FN property   
$statement = $vCard->getProperty($vcard\_FN);  
$value = $statement->getObject();

In this example, the vcard resource has only one vcard:FN property. RDF permits
a resource to have several properties of the same name; for example Adam might
have more than one nickname. Let's give him two:

// Add two nickname properties to vcard  
$literal1 = $model->createLiteral("Smithy");  
$literal2 = $model->createLiteral("Adman");   
$vCard->addProperty($vcard\_NICKNAME, $literal1);  
$vCard->addProperty($vcard\_NICKNAME, $literal2);

Calling $vcard->getProperty(VCARD.NICKNAME) will return one of the values,
but it is indeterminate which one. So if it is possible that a property might
occur more than once, then the $resource->listProperties($property) method
has to be used to get an array containing all statements with this resource and property:

// List the nicknames  
echo '<b>Known nicknames for '.$fullNameLiteral->getLabel().':</b><BR>';  
foreach ($vCard->listProperties($vcard\_NICKNAME) as $currentResource)   
{   
    echo $currentResource->getLabelObject().'<BR>';  
};

## Querying a Model

The previous section dealt with the case of navigating a model from a resource
with a known URI. This section deals with searching a model. RAP's ResModel
API only supports a limited query primitive. More powerful query facilities
are provided through the RDQL query language which is described in a separate
tutorial.

$model->listSubjects() returns an iterator over all resources that have
properties, *ie*  are the subject of some statement. $model->listSubjectsWithProperty($property,
$value) will return an iterator over all the resources which have a property
$property with value $value.

Example: Iterate over all vcards which having FN property:

// Iterate over all vcards which having FN property  
$iter = $model->listSubjectsWithProperty(new ResResource(VCARD\_NS.'FN'));
  
for ($iter->rewind(); $iter->valid(); $iter->next())  
{  
    $currentResource=$iter->current();  
};

## Containers

RDF defines a special kind of resources for representing collections of things.
These resources are called *containers*. The members of a container can
be either literals or resources. There are three kinds of container:

- a BAG is an unordered collection
- an ALT is an unordered collection intended to represent alternatives
- a SEQ is an ordered collection

A container is represented by a resource. That resource will have an rdf:type property whose value should be one of rdf:Bag, rdf:Alt or rdf:Seq, or a subclass of one of these, depending on the type of the container. The first member of the container is the value of the container's rdf:\_1 property; the second member of the container is the value of the container's rdf:\_2 property and so on. The rdf:\_nnn properties are known as the *ordinal properties* .

For example, the Model for a simple bag containing the vcards of the Smith's might look like this:

Whilst the members of the bag are represented by the properties rdf:\_1, rdf:\_2 etc the ordering of the properties is not significant. We could switch the values of the rdf:\_1 and rdf:\_2 properties and the resulting Model would represent the same information.

Whilst containers can be handled using the basic machinery of
resources and properties, RAP has explicit implementation classes to handle
them.

// Create a bag   
$bag\_smiths = $model->createBag();

$beckySmith = $model->createResource('http://somewhere/BeckySmith');  
$beckySmithFN = $model->createLiteral('Becky Smith');  
$beckySmith->addProperty($vcard\_FN,$beckySmithFN );

// Add persons to bag  
$bag\_smiths->add($beckySmith);  
$bag\_smiths->add($johnSmith);

If we serialize this Model, you'll get something like this:

<rdf:Description rdf:nodeID="A3">   
    <rdf:type rdf:resource='http://www.w3.org/1999/02/22-rdf-syntax-ns#Bag'/>   
    <rdf:\_1 rdf:resource='http://somewhere/BeckySmith'/>   
    <rdf:\_2 rdf:resource='http://somewhere/JohnSmith'/>   
</rdf:Description>   

which represents the Bag resource.

The container implementation provides a getMembers() method for getting the
content of a container, as an array of ResResources:

// Print out the full names of the members of the bag  
echo '<BR><BR><b>Print out the full names of the members of the bag:</b></BR>';   
foreach ($bag\_smiths->getMembers() as $resResource)  
{  
    // Retrieve the value of the FN property   
    $statement = $resResource->getProperty($vcard\_FN);  
    echo $statement->getLabelObject().'<BR>';   
};

The RAP ResContainer classes currently ensure that the the list of ordinal
properties starts with rdf:\_1 and is contiguous. The RDFCore WG have relaxed
this constrain, so RAPS's container implementation might follow in the future.

It is not a problem to use a container object and to modify the state underlying
model using lower level methods at the same time, because all container level
operations are directly written into the underlying model.

Write the model as HTML table:

echo '<BR><BR>All Statements as HTML table';  
$model->writeAsHTMLTable();
